# Enhancement of Cholinesterase Inhibition of *Alpinia galanga* (L.) Willd. Essential Oil by Microemulsions

**DOI:** 10.3390/molecules27103275

**Published:** 2022-05-19

**Authors:** Wantida Chaiyana, Suwannee Sriyab, Siriporn Okonogi

**Affiliations:** 1 Department of Pharmaceutical Sciences, Faculty of Pharmacy, Chiang Mai University, Chiang Mai 50200, Thailand; wan.su@hotmail.com (S.S.); siriporn.okonogi@cmu.ac.th (S.O.); 2 Research Center of Pharmaceutical Nanotechnology, Chiang Mai University, Chiang Mai 50200, Thailand; 3 Innovation Center for Holistic Health, Nutraceuticals and Cosmeceuticals, Faculty of Pharmacy, Chiang Mai University, Chiang Mai 50200, Thailand

**Keywords:** *Alpinia galanga*, Alzheimer’s disease, acetylcholinesterase, butyrylcholinesterase, selectivity, microemulsion

## Abstract

This study aimed to investigate the chemical composition and reveal the selective inhibitory activity of *Alpinia galanga* (L.) Willd. essential oil (AGO) on acetylcholinesterase (AChE) compared to butyrylcholinesterase (BChE). The chemical composition of AGO was investigated by means of gas chromatography–mass spectrometry. Ellman’s method was used to determine the inhibitory activities against AChE and BChE. Microemulsion systems with desirable anticholinesterase effects were developed. Methyl cinnamate and 1,8-cineole were reported as the major component of AGO. The IC_50_ values of *A. galanga* oil against AChE and BChE were 24.6 ± 9.6 and 825.4 ± 340.1 µg/mL, respectively. The superior selectivity of AGO on AChE (34.8 ± 8.9) compared to galantamine hydrobromide (6.4 ± 1.5) suggested AGO to be an effective ingredient with fewer side effects for Alzheimer’s treatment. Interestingly, the microemulsion of AGO possessed significantly higher anticholinesterase activity than that of native oil alone. Therefore, microemulsion of AGO is a promising alternative approach for the treatment of Alzheimer’s disease.

## 1. Introduction

*Alpinia galanga* (L.) Willd., belonging to the family *Zingiberaceae*, is a medicinal plant indigenous to China, India and Southeast Asian countries. The rhizomes of this plant are extensively used for flavoring food and are also used in traditional medicine for various ailments [1]. It is used in Asian traditional medicine as an antiseptic, antibacterial, and carminative agents, as a digestive stimulant, and for treating diabetes mellitus [2,3,4]. In China, *A. galanga* is used as a folk medicine for stomach conditions [3]. In India, the plant is a reputed drug in indigenous systems of medicine, and is widely used in southern India as a domestic remedy [5]. The plant is used in dyspepsia, fevers, incontinence of urine, treatment of halitosis, and reduction of voice hoarseness during throat infections [6]. In Thailand, the rhizomes are used as carminative, anti-flatulence, antifungal, and anti-itching agents [7]. In Malaysia, the rhizomes are used for the treatment of coughs, asthma, bronchitis, headache, inflammation, rheumatoid arthritis, and colic [8]. Recent studies have indicated the pharmacological properties of *A. galanga*, including antitumor [9], antibacterial [10,11], anti-ulcer [12], antifungal [13], inhibition of HIV 1 replication [14], and anticholinesterase effects [15,16,17].

Our previous study demonstrated the anticholinesterase activities of twenty-five essential oils obtained from different plants, and the essential oil from *A. galanga* rhizome (AGO) showed potent acetylcholinesterase (AChE) inhibition [16]. Therefore, further investigations on the dose–response relationship, AChE selectivity, and safety profile of AGO are of interest, and are worth pursuing. The main function of AChE is the rapid hydrolysis of the neurotransmitter acetylcholine (ACh) on the cholinergic synapses associated with Alzheimer’s disease (AD), whereas butyrylcholinesterase (BChE) is not correlated with any physiological abnormalities [18,19,20]. BChE has been suggested to function as an embryonic acetylcholinesterase [21], and it is important to pharmacology and toxicology, since it hydrolyzes ester-containing drugs and scavenges cholinesterase inhibitors, including potent organophosphorus nerve agents, before they reach their synaptic targets [22]. AChE is known to be abundant in the brain, muscle, and erythrocyte membrane, whereas BChE has higher activity in the liver, intestine, heart, kidney, and lung [23,24]. Although AChE levels are decreased by up to nearly 85% in specific brain regions in AD patients, with this amount rising with disease progression [25], the presence of BChE in many important organs might be due to severe side effects. Because of the predominant AChE inhibition of AGO, it might be a promising approach for the treatment of AD, offering good tolerance and a reduction in the BChE effect.

The high selectivity and electrostatic charge of blood–brain barrier have hindered the treatment of brain disorders, so many attempts have been made and strategies continue to be developed for achieving successful symptomatic therapy, preventing the progression of many types of neurodegenerative disorders, and minimizing the well-known severe consequences of therapeutic medicines [26]. Nanotechnology has been suggested for use in neuroscience to treat neurodegenerative disorders [27]. Among various types of nano delivery systems, microemulsions were developed to improve brain targeting of various compounds, e.g., Tracine [28], Donepezil [29], piperine [30], huperzine A and ligustrazine phosphate [31], morin hydrate [32], etc., when administered through various routes, including orally, transdermally, and intranasally. Microemulsions, which are spontaneously formed systems that increase drug solubility, are easily produced on the manufacturing scale and have excellent thermodynamic stability, have been widely researched as a delivery system due to their significant potential to improve the transport of a wide spectrum of medicinal compounds through biological membranes [28,29,30,31,32]. Aside from increasing the rate of drug penetration across the blood–brain barrier, resulting in higher drug concentrations at the target site, microemulsions can eventually lead to a decrease in unfavorable side-effects due their site-specific delivery to the brain [32].

To this end, this study aims to investigate the dose–response effect of AGO against AChE and BChE. The selectivity for AChE is then determined. Although *A. galanga* has been known to be an edible plant since ancient times, its toxicity toward peripheral blood mononuclear cells (PBMCs) was investigated in order to confirm the safety of using AGO. Moreover, we evaluated the incorporation of AGO in a novel topical delivery system like a microemulsion (ME), which is an ideal therapeutic approach for chronic neurological disorders in elderly people, especially in AD [33]. The optimum HLB value for ME formation was evaluated. Factors influencing the ME region in the pseudoternary phase diagram that were investigated include co-surfactant type and surfactant/co-surfactant ratio. Suitable systems were finally derived for the anticholinesterase activities and AChE selectivity.

## 2. Materials and Methods

### 2.1. Plant Material

*Alpinia galanga* Linn. was purchased from local market in Chiang Mai, Thailand. The plant was authenticated and voucher specimens with the number 22124 were deposited in the Herbarium of the Faculty of Pharmacy, Chiang Mai University, Thailand.

### 2.2. Chemicals and Enzymes

Acetylcholinesterase (AChE, specific activity 425.94 U/mg) from *Electrophorus electricus*, butyrylcholinesterase (BChE, specific activity 7.4 U/mg) from equine serum, acetylthiocholine iodide (ATCI), butyrylthiocholine iodide (BTCI), galantamine hydrobromide, 5,5′-dithiobis (2-nitrobenzoic acid) (DTNB), propylene glycol, glycerin, polyethylene glycol 400 (PEG 400), polyethylene glycol 600 (PEG 600), polysorbate 20 (Tween^®^ 20), and sorbitane monooleate (Span^®^ 80) were analytical grade and were purchased from Sigma-Aldrich Chemicals (St. Louis, MO, USA). Ethanol and propan-2-ol were analytical grade and purchased from Merck (Darmstadt, Germany).

### 2.3. Extraction of AGO

Fresh *A. galanga* rhizomes were chopped into small pieces and subjected to hydro-distillation for 3 h using a Clevenger-type apparatus. AGO extract was collected and stored in a refrigerator, away from light, until it was further used.

### 2.4. GC-MS Analysis

AGO was injected into GC-MS in order to evaluate the essential oil’s chemical constituents. The GC-MS analysis was carried out on an Agilent 6890 GC (Agilent Technologies, Santa Clara, CA, USA) coupled to an electron impact (EI, 70 eV) HP 5973 mass selective detector (Hewlett Packard, Palo Alto, CA, USA) and fitted with a fused silica capillary column (HP-5MS) supplied by Hewlett Packard, Palo Alto, CA, USA (30.0 m × 250 mm, i.d. 0.25 mm film thickness). Helium was used as a carrier gas with a flow rate of 1.0 mL/min. The injector temperature was set at 260 °C, whereas the oven temperature was set at 100 °C and isothermally held for 3 min. Thereafter, the temperature was increased to 188 °C at a rate of increase of 3 °C/min, followed by increasing the temperature to 280 °C with rate of increase of 20 °C/min. Finally, the temperature was isothermally maintained at 280 °C for 3 min. A detector’s temperature was also set to 280 °C. No peaks were detected below 100 °C following the initial injection. The identification of compounds was performed based on the Wiley/NIST database. The content of each component was calculated based on the total area under the curve of all peaks obtained from the tested sample. An aliquot of *n*-alkanes (*n*-C10 to *n*-C28) mixture was used as the reference standard for calculating Kováts retention indices (RI) using the following equation [34]:

(1) $$RI=100Z+100\left(\frac{\log\;tR\left(x\right)-\;\log\;tR\left(Z\right)}{\log\;tR\left(Z+1\right)-\;\log\;tR\left(Z\right)}\right),$$

where *RI* is the retention index of compound *x*, *Z* is the number of carbon atoms of the *n*-alkane eluted before compound *x*, *Z +* 1 is the number of carbon atoms of the *n*-alkane eluted after compound *x*, *tR(x)* is the retention time of compound *x*, *tR(Z)* is the retention time of the *n*-alkane eluted before compound *x*, *tR(Z* + 1*)* is the retention time of *n*-alkane eluted after compound *x*.

### 2.5. Cholinesterase Activity Determination

Ellman’s method was used to determine the cholinesterase enzyme inhibitory activities [35,36]. Electric eel AChE and horse serum BChE were used as cholinesterase enzymes, along with their own substrates, which were ATCI and BTCI, respectively. Briefly, 50 mM Tris-HCl buffer pH 8.0, 1.5 mM substrate solution, 3 mM DTNB, and the tested sample dissolved in Tris-HCl buffer containing 10% methanol were mixed in the ratio 2:1:5:1, respectively. Thereafter, 0.25 U/mL AChE or 0.91 U/mL BChE was added, and the reaction was spectrophotometrically evaluated for 120 s at 415 nm using a microplate reader (Bio-Rad Laboratories Ltd., Tokyo, Japan). Prior to each test, the enzyme activities were verified and accepted only when they were greater than 90%. The Tris-HCl buffer containing 10% methanol, which was used as a solvent for dissolving the tested sample, was used as negative control for the evaluation of cholinesterase activity. In the case of the evaluation of the inhibitory activity of the ME, 25 μL of ME containing 10% of AGO was added instead of AGO in Tris-HCl buffer with 10% methanol. Therefore, there was no additional methanol, which would disrupt the ME structure. The mixture containing 10% methanol, 14% PG, and 56% Tween^®^ 20 was used as a negative control in this case. Galantamine hydrobromide was used as a positive control. The experiments were performed in triplicate. The slope of the plot of absorbance versus time was taken as the enzymatic reaction rate. The enzyme inhibitory activity was calculated using the following equation:

(2) $$\%\;\mathrm{Inhibition}=\left(\frac{Vs-Vb}{Vb}\right)\times 100$$

where *Vs* is the reaction rate in the presence of AGO and *Vb* is the reaction rate in the absence of AGO. IC_50_ values were statistically evaluated using the Graphpad/Prism program. The AChE selectivity index (*SI*) was defined using the following equation:

(3) $$SI=\frac{IC_{50\;BChE}}{IC_{50\;AChE}}$$

where *SI* is the AChE selectivity index and *IC*_50 *BChE*_ is the concentration of the test compound required to inhibit BChE activity by 50%, and *IC*_50 *AChE*_ is the concentration of the test compound required to inhibit AChE activity by 50%.

### 2.6. Cytotoxicity

The effect of AGO on the cell viability of peripheral blood mononuclear cells (PBMCs) was determined by means of MTT assay, as described by Chaiyana et al. [37], with some modifications, as described below.

#### 2.6.1. PBMC Isolation

Blood (20–25 mL) of normal volunteers was taken from the same donors throughout the research using a 25 mL syringe. The blood samples were diluted with the same volume of phosphate buffer saline (PBS). After that, the diluted blood samples were carefully layered on Ficoll-Paque Plus. Then, the mixture was centrifuged under 5000× *g* for 30 min at 18–20 °C. The undisturbed lymphocyte layer was carefully transferred out. The lymphocyte was washed and pelleted down with three volumes of PBS for twice and resuspended RPMI-1640 media with 100 IU/mL of penicillin, 100 μg/mL of streptomycin, 10%, *v/v* fetal bovine serum (FBS). Cell counting was performed to determine the PBMC cell number with equal volume of trypan blue.

#### 2.6.2. Cell Viability Assay

In brief, 100 μL of PBMC with cell concentration at 105 cells/mL was added into all wells in the 96-well plate and incubated in an incubator at 37 °C, 5% CO_2_ and 90% humidity for 24 h. Then, 100 μL of various concentrations of the extract was added to the cells compared with untreated cells and incubate again in the same condition for 48 h. After the corresponding period, 100 μL of media was removed from each well, and 25 μL of MTT at 5 mg/mL was added to each well before a further 4 h incubation. All the media was removed by turning the 96-well plate upside down. Then, 200 μL of dimethyl sulfoxide (DMSO) was added to each well to extract and solubilize the formazan crystal. Finally, the plate was read at 540 nm by using microplate reader. All experiments were performed in triplicate. The percentage cell viability was calculated using the following equation:

(4) $$\%\;\mathrm{Cell}\;\mathrm{viability}=\left(\frac{ODs-ODb}{ODc-ODb}\right)\times 100$$

where *ODs* is the OD value of the well treated with a certain concentration of AGO, *ODc* is the OD value of the well in the absence of AGO, and *ODb* is the OD value of the blank.

### 2.7. Optimum HLB Value Determination

Tween^®^ 20 (HLB = 16.7) was mixed with Span^®^ 80 (HLB = 4.3) in various ratios to produce surfactant combinations with different HLB values ranging from 4.3 to 16.7. Various formulations containing 10% AGO, 20% water, 56% surfactant mixture (Tween^®^ 20 and/or Span^®^ 80), and 14% co-surfactant were formulated. The formulations were characterized as ME when they met all of the physicochemical criteria for a microemulsion, including thermodynamically stable microemulsion, which include being formed spontaneously, being transparent, possessing low viscosity, and being optically isotropic [38]. The characterizations of their transparency and low viscosity were performd by visual inspection. A polarizing light microscope was used to assess the isotropy of each formulation (Motic BA310 POL, Innovation Way, Carlsbad, CA, USA).

### 2.8. Photon Correlation Spectroscopy

Measurement of internal droplet size of the monophasic systems containing 10% AGO, 20% water, 56% surfactant, and 14% co-surfactant were carried out using photon correlation spectroscopy (Zetasizer^®^ version 5.00, Malvern Instruments Ltd., Malvern, UK) at a fixed angle of 173°. The results are expressed as the mean ± standard deviation (SD) of at least ten measurements of one sample.

### 2.9. Construction of Phase Diagrams

A water titration method was used to construct the pseudoternary phase diagrams [36]. To generate a surfactant combination (Smix), a surfactant (Tween^®^ 20) was combined with a co-surfactant (ethanol, propan-2-ol, glycerin, PG, PEG 400, or PEG 600) at weight ratios of 4:1, 1:1, or 1:4. The essential oils and Smix were then blended in different weight ratios. Thereafter, the resulting mixes were titrated with water at room temperature under gentle agitation. When the samples presented visually as clear liquids, they were classified as ME. The formulations were generated in triplicate. OriginPro 8 was used to construct the pseudoternary phase diagrams.

### 2.10. Development of Microemulsion

The ingredients of the microemulsion were chosen from the pseudoternary phase diagram with the largest microemulsion area. The lowest possible concentration of Smix was used when the AGO was fixed at 10%. The selected microemulsion was formed spontaneously after mixing all ingredients together. The formulation was then characterized with respect to its internal droplet size and PDI using photon correlation spectroscopy (Zetasizer^®^ version 5.00, Malvern Instruments Ltd., Malvern, UK) at a fixed angle of 173°. A polarizing light microscope was used to assess the isotropy of the microemulsion (Motic BA310 POL, Innovation Way, Carlsbad, CA, USA). The microemulsion was investigated with respect to its physical stability under heating–cooling conditions. After keeping the microemulsion in a well-closed container at 4 °C (24 h) and 45 °C (24 h) for 8 cycles, it was characterized with respect to internal droplet size, PDI, and isotropy following the methods described above.

### 2.11. Statistical Analysis

All data are presented as a mean ± SD. The One-Way ANOVA: post hoc test was used to assess individual differences. *p* < 0.05 denotes significance in all circumstances.

## 3. Results

### 3.1. Yield, Density, and GC-MS Analysis of AGO

Hydrodistillation of *A. galanga* yielded a light yellowish AGO with a yield of 0.14% *v*/*w* and a density of 0.9 g/mL. Twenty-seven constituents, making up 93.06% of the total AGO composition, were identified on the basis of a comparison of their mass spectra with those stored in the Wiley/NIST Mass Spectral Library. Further identification of AGO components was achieved by comparing their *RI* with those in the literatures [39,40,41,42,43,44,45,46,47,48]. The relative concentrations of the volatile components identified are presented in Table 1 on the basis of their elution order on the HP-5 column. The main constituents of AGO (concentrations higher than 3%) were methyl cinnamate (33.31%), 1,8-cineole (29.64%), germacrene D (5.62%), *p*-cymen-8-ol (3.06%), and *α*-amorphene (3.01%). The results were in a good agreement with previous reports that 1,8-cineole and methyl cinnamate are the major constituents of AGO [48,49]. However, other variations in their chemical compositions were found, which were attributed to variations in their environmental conditions and geographical origin, significantly affecting the chemical compositions of the essential oils [50]. Although the area under the curve or the height of each peak in the GC chromatogram represent the relative amounts of the chemical constituents, a validated quantitative analysis is also suggested in future investigations.

### 3.2. Cholinesterase Inhibition of AGO

**Figure 1:**
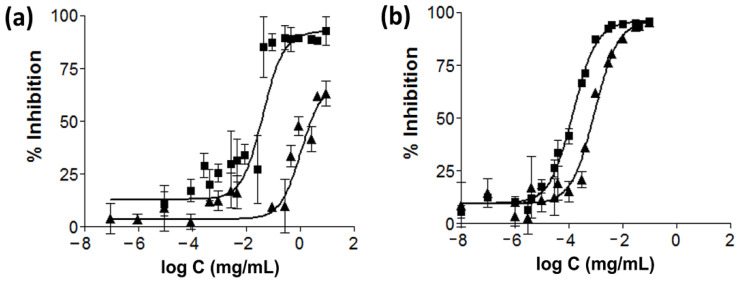
Dose–response curves for anti-AChE (■) and anti-BChE (▲) activities of AGO (**a**) and galantamine hydrobromide (**b**).

The dose–response curves for the anti-AChE and anti-BChE activity of AGO are shown in Figure 1a and compared with galantamine hydrobromide in Figure 1b. The calculated IC_50_ values show that AGO can be characterized as a moderate AChE inhibitor, with an IC_50_ value of 24.56 ± 9.55 µg/mL, and a weak BChE inhibitor with an IC_50_ value of 825.37 ± 340.14 µg/mL, compared to galantamine hydrobromide, which is a potent inhibitor of AChE and BChE, with an IC_50_ value against AChE of 0.14 ± 0.02 µg/mL and an IC_50_ value against BChE of 0.94 ± 0.34 µg/mL. The calculated *SI* values of AGO and galantamine hydrobromide were 34.8 ± 8.9 and 6.4 ± 1.5, respectively. A relevant point of comparison to these results are the results presented in a previous study by Zhao et al., who reported an *SI* value of galantamine of 9.13 [51]. The significantly higher *SI* indicates the higher selectivity of AGO (*p* < 0.05). Although AGO was significantly more selective for AChE than galantamine hydrobromide, its activity was approximately 175 times lower. As a result, a higher dosage of AGO would be necessary; nevertheless, its selectivity for AChE would result in better patient compliance due to the lesser extent of the side effects. From the point of view of adverse events, a specific AChE inhibitor (donepezil) is tolerated best, and gradual dose increments in order to prevent adverse events are necessary for selective inhibitors (galantamine) and dual inhibitors (rivastigmine) [52]. Therefore, AGO, which can be classified as being AChE selective, might be a promising and effective treatment strategy for AD patients, providing better tolerance. However, further in vivo experiments and clinical studies should be performed.

### 3.3. Safety Profile of AGO on Normal Cells

**Figure 2:**
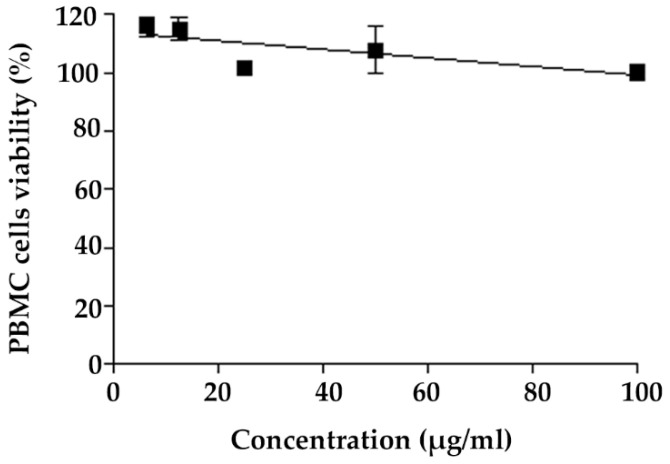
Dose–response curves of viability of PBMC versus concentration of AGO.

PBMCs were used as a representative of normal cells to investigate the safety of AGO. The dose–response curve of PBMC viability is shown in Figure 2. The results indicate that cell viability was around 100% at AGO concentrations of up to 100 µg/mL, indicating the safety of AGO. The ability of a biomaterial to keep PBMC viability unaltered is a known method for evaluating their safety [53].

### 3.4. Optimum HLB Value Determination

**Figure 3:**
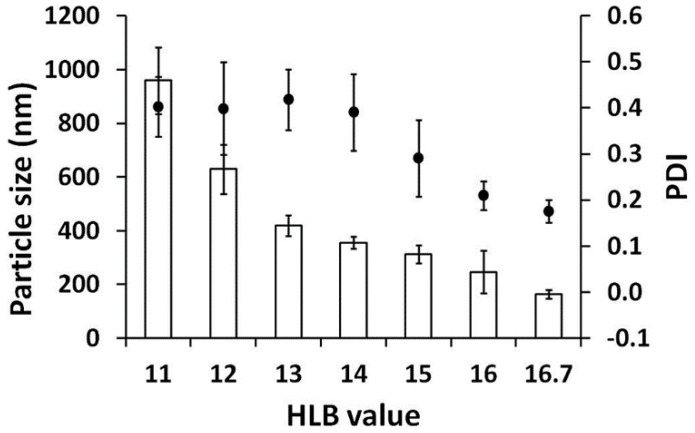
Internal droplet size (☐) and PDI (●) of AGO/surfactant/co-surfactant/water system. Effect of HLB values of surfactants was studied with PG as a co-surfactant.

Two surfactants (Tween^®^ 20 and Span^®^ 80) were mixed in various ratios to produce the desired HLB values. Formulations with surfactant combinations with HLB values ranging from 4.3 to 10 were turbid, and phase separation was found, whereas formulations with higher HLB values (higher than 11) were isotropic transparent liquids, which were optically isotropic without any birefringence under the polarizing light microscope. The internal droplet sizes of the clear formulations are shown in Figure 3. Droplet size decreased with higher values of HLB, and the smallest droplet size was obtained for a HLB value of 16.7. Therefore, Tween^®^ 20 with an HLB value of 16.7 is the most suitable surfactant for ME formation of AGO. These results are in a good agreement with a previous study investigating the effect of surfactants with HLB values ranging from 10 to 16, which suggested that ME was more easily formed at higher HLB values and when the ME region covered a larger area for more polar surfactants [54,55].

Furthermore, another study by Ban et al., investigating the effects of different HLB values (9.8 to 12.8) obtained by mixing different poly(oxyethylene) hydrogenated castor oil surfactants on emulsion formation, reported that the smallest emulsion droplets were formed with the highest HLB value of 12.8 [56].

### 3.5. Developement of AGO Microemulsions

**Figure 4:**
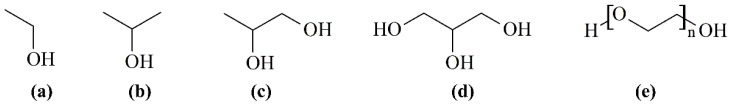
Chemical structures of ethanol (**a**), propan-2-ol (**b**), PG (**c**), glycerin (**d**), and PEG (**e**).

**Figure 5:**
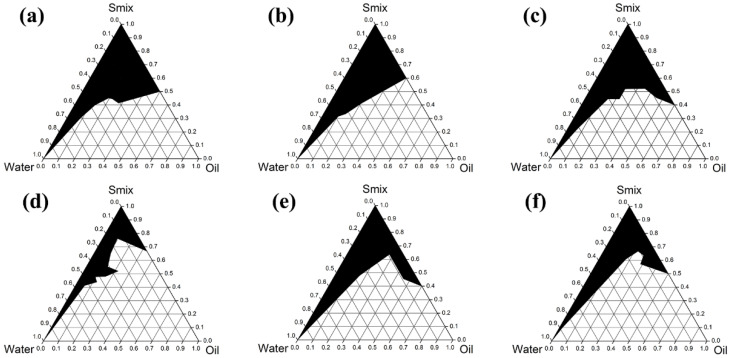
Pseudoternary phase diagrams of AGO/Tween^®^ 20/co-surfactant/water when the co-surfactants were ethanol (**a**), propan-2-ol (**b**), PG (**c**), glycerin (**d**), PEG 400 (**e**) and PEG 600 (**f**).

Six co-surfactants (Figure 4) were used to investigate the effect of co-surfactants on the phase diagram of ME. As can be seen in Figure 5, the largest ME region was that for PG, followed by ethanol, propan-2-ol, PEG 400, PEG 600, and glycerin, with areas of 35.4%, 34.2%, 33.1%, 30.2%, 27.6%, and 17.8%, respectively. These results indicate that there is no relationship between the ME region and number of hydroxyl groups or hydrocarbon chain length. However, the relationships between the ME region and the dielectric constant (Table 2) are dominant. Co-surfactants with dielectric constants in the range of 22 to 32 were appropriated for use with Tween^®^ 20 to form MEs. These results are in line with the expectations presented in a previous paper [57], since the semipolar co-surfactants act as intermediate solvents to improve the miscibility of polar and nonpolar liquids. PG, which provided the largest ME region, was selected for the study of surfactant/co-surfactant ratios. Higher proportions of surfactant in Smix provided a larger ME region, as shown in Figure 5.

**Figure 6:**
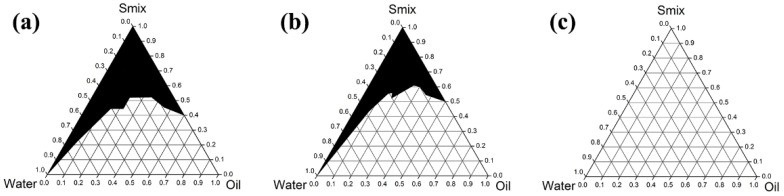
Pseudoternary phase diagrams of AGO/Tween^®^ 20/PG/water when the Tween^®^ 20/PG ratios were 4:1 (**a**), 1:1 (**b**), and 1:4 (**c**).

**Figure 7:**
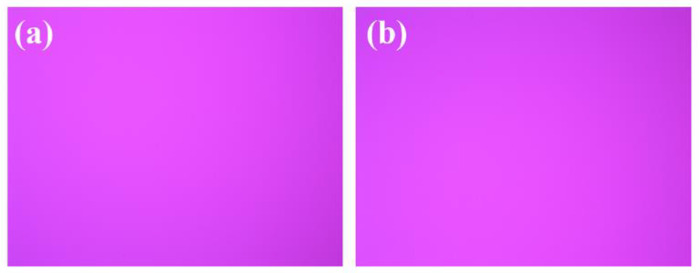
Microemulsion of AGO/Tween^®^ 20/PG/water system was isotropic under polarizing light microscope before (**a**) and after (**b**) the stability test under heating–cooling conditions. The magnification was 10×.

The pseudoternary phase diagram of the AGO/Tween^®^ 20/PG/water system exhibited the largest microemulsion region when the ratio of Tween^®^ 20 and PG was 4:1 (Figure 6a). Therefore, these ingredients were used for microemulsion development. When 10% *w*/*w* of AGO (equivalent to 100 µg/mL) was used, the lowest Smix concentration required for the microemulsion was 30% *w*/*w* (24% Tween^®^ 20 and 6% PG). The obtained formulation was a spontaneously formed transparent liquid with excellent stability. The internal droplet size (71.6 ± 3.4 nm) and PDI (0.217 ± 0.012) did not change after reaching stability under heating–cooling conditions (Table 3). The isotropy of the microemulsion was detected under polarizing light microscope, since no birefringence was observed (Figure 7).

### 3.6. Cholinesterase Inhibition of AGO ME

The IC_50_ values of the AGO/Tween^®^ 20/PG/water system against AChE and BChE were compared with that of native AGO alone. Other components, Tween^®^ 20 and PG, were also investigated. The results are shown in Table 4. With equal concentrations of AGO (100 µg/mL), the inhibitory activities of ME were significantly higher than those of AGO alone against both AChE and BChE (*p* < 0.05). Tween^®^ 20 and PG showed no inhibition against either enzyme. Therefore, it was considered that loading AGO in a monophasic ME system could greatly enhance the inhibition of the enzymes by increasing the aqueous solubility and compatibility with the test solution in Ellman’s assay, since the cholinesterase inhibitory activity of AGO was hindered due to the poor aqueous solubility.

## 4. Conclusions

The present research work reports the greater selectivity of AGO with respect to AChE over BChE, with an *SI* value of 34.8, leading to a promising alternative approach for treating AD entailing fewer severe side effects. AGO was characterized as a moderate AChE inhibitor with the IC_50_ value of 24.56 ± 9.55 µg/mL. The safety profile of AGO was investigated, and it was demonstrated to be safe, since PBMC cell viability was not altered after exposure to 100 µg/mL AGO. The present study also explored suitable transdermal ME of AGO for achieving the desired anticholinesterase effects. The optimum HLB value for ME formation was 16.7, which was derived from Tween^®^ 20. PG was the most suitable co-surfactant, providing the highest ME region in the phase diagram. Surfactant-to-co-surfactant ratio affects the ME region dramatically, and 4:1 was found to be the best ratio. ME systems composed of 10% *w*/*w* AGO, 24% *w*/*w* Tween^®^ 20, 6% *w*/*w* PG, and 50% *w*/*w* DI water showed significantly higher anticholinesterase activity than those consisting of AGO alone. Therefore, the results of this study suggest the successful development of ME of AGO, and that it might have potential as a treatment for patients suffering from AD. The transdermal or intranasal use of AGO is proposed as a prospective future investigation.

## Figures and Tables

**Table 1:** Volatile constituents of *A. galanga* rhizome.

Peak	RT	Compound	%Area	Identification	RI_calc_	RI_literature_ [Ref]
1	3.32	1,8-Cineole	**29.64**	MS, RI	1034	1034 [39,40,41]
2	3.56	*γ*-Terpinene	1.22	MS, RI	1057	1057 [42]
3	3.99	*α*-Terpinolene	0.44	MS, RI	1093	1088 [41,43,44]
4	4.09	1-Undecene	0.20	MS, RI	1100	1100 [45]
5	5.48	(-)-Borneol	0.72	MS, RI	1160	1165 [43,44]
6	6.03	*p*-Cymen-8-ol	**3.06**	MS, RI	1179	1179 [42]
7	6.25	*α*-Terpineol	0.20	MS, RI	1186	1189 [39,40,43,44]
8	7.5	*Z*-Citral	1.23	MS, RI	1240	1240 [39]
9	8.51	(-)-Bornyl acetate	0.39	MS, RI	1284	1284 [39,46]
10	10.56	Methyl cinnamate	**33.31**	MS, RI	1347	1352 [47]
11	10.78	*α*-Cubebene	0.15	MS, RI	1352	1352 [39,40,42]
12	11.55	Decaoic acid	1.31	MS, RI	1371	1367 [45]
13	12.3	*β*-Elemene	1.91	MS, RI	1388	1387 [39,40,42]
14	12.83	*α*-Gurjunene	0.20	MS, RI	1400	1405 [47]
15	13.39	*β*-Caryophyllene	3.38	MS, RI	1416	1415 [39,40]
16	15.44	*β*-Farnesene	0.42	MS, RI	1469	1463 [47,48]
17	15.56	*β*-Selinene	0.46	MS, RI	1472	1475 [39,42]
18	15.71	*δ*-Selinene	0.31	MS, RI	1476	1491 [41]
19	15.95	Germacrene D	**5.62**	MS, RI	1482	1483 [42,47]
20	16.5	*α*-Amorphene	**3.01**	MS, RI	1494	1487 [40]
21	16.65	*α*-Selinene	0.83	MS, RI	1498	1498 [39]
22	16.79	*γ*-Bisabolene	2.25	MS, RI	1511	1510 [40]
23	21.08	*α*-Cadinol	0.61	MS, RI	1640	1640 [39,40]
24	21.62	*τ*-Muurolol	0.40	MS, RI	1645	1641 [42]
25	22.21	*nor*-Copaanone	0.84	MS, RI	1650	1622 [42]
26	22.45	Dillapiol	0.65	MS, RI	1652	1628 [46]
27	27.46	*α*-Bergamotol	0.30	MS, RI	1691	1693 [44]

**Note:** The major compounds with the amount above 5% are bolded.

**Table 2:** Dielectric constant (δ) of co-surfactant at 20 °C.

Co-Surfactants	Dielectric Constant
Propylene glycol	32.1
Ethanol	25
Propan-2-ol	22
Polyethylene glycol 400	12.4
Polyethylene glycol 600	10.2
Glycerin	46

**Table 3:** Internal droplet size and PDI of AGO microemulsion before and after the stability test.

Characterizations	Stability Test	
	Before	After
Internal droplet size (nm)	71.6 ± 3.4	74.1 ± 2.8
PDI	0.217 ± 0.012	0.224 ± 0.014

**Table 4:** Anticholinesterase activities of microemulsion formulation and each component.

Formulation/Component	% Inhibition	
	AChE	BChE
AGO (100 µg/mL)/Tween^®^ 20/PG/water	95.63 ± 7.10	14.07 ± 1.44
AGO (100 µg/mL)	34.31 ± 8.08	1.40 ± 2.43
Tween^®^ 20 (0.1 mg/mL)	−0.03 ± 0.48	−0.04 ± 0.20
PG (0.1 mg/mL)	−1.65 ± 2.77	−0.35 ± 1.24

## Data Availability

The data presented in this study are available on request from the corresponding author.
